# Mitigation of Greenhouse Gas Emissions from Rice via Manipulation of Key Root Traits

**DOI:** 10.1186/s12284-023-00638-z

**Published:** 2023-05-10

**Authors:** Juan de la Cruz Jiménez, Ole Pedersen

**Affiliations:** 1 Department of Biology, University of Copenhagen, Universitetsparken 4, 3rd floor, Copenhagen, 2100 Denmark; 2 School of Agriculture and Environment, The University of Western Australia, 35 Stirling Highway, Crawley, WA 6009 Australia

**Keywords:** Aerenchyma, barrier to radial O_2_ loss, oxidation, CH_4_, CO_2_, N_2_O

## Abstract

Rice production worldwide represents a major anthropogenic source of greenhouse gas emissions. Nitrogen fertilization and irrigation practices have been fundamental to achieve optimal rice yields, but these agricultural practices together with by-products from plants and microorganisms, facilitate the production, accumulation and venting of vast amounts of CO_2_, CH_4_ and N_2_O. We propose that the development of elite rice varieties should target root traits enabling an effective internal O_2_ diffusion, via enlarged aerenchyma channels. Moreover, gas tight barriers impeding radial O_2_ loss in basal parts of the roots will increase O_2_ diffusion to the root apex where molecular O_2_ diffuses into the rhizosphere. These developments result in plants with roots penetrating deeper into the flooded anoxic soils, producing higher volumes of oxic conditions in the interface between roots and rhizosphere. Molecular O_2_ in these zones promotes CH_4_ oxidation into CO_2_ by methanotrophs and nitrification (conversion of NH_4_^+^ into NO_3_^-^), reducing greenhouse gas production and at the same time improving plant nutrition. Moreover, roots with tight barriers to radial O_2_ loss will have restricted diffusional entry of CH_4_ produced in the anoxic parts of the rhizosphere and therefore plant-mediated diffusion will be reduced. In this review, we describe how the exploitation of these key root traits in rice can potentially reduce greenhouse gas emissions from paddy fields.

## Introduction

**Fig. 1:**
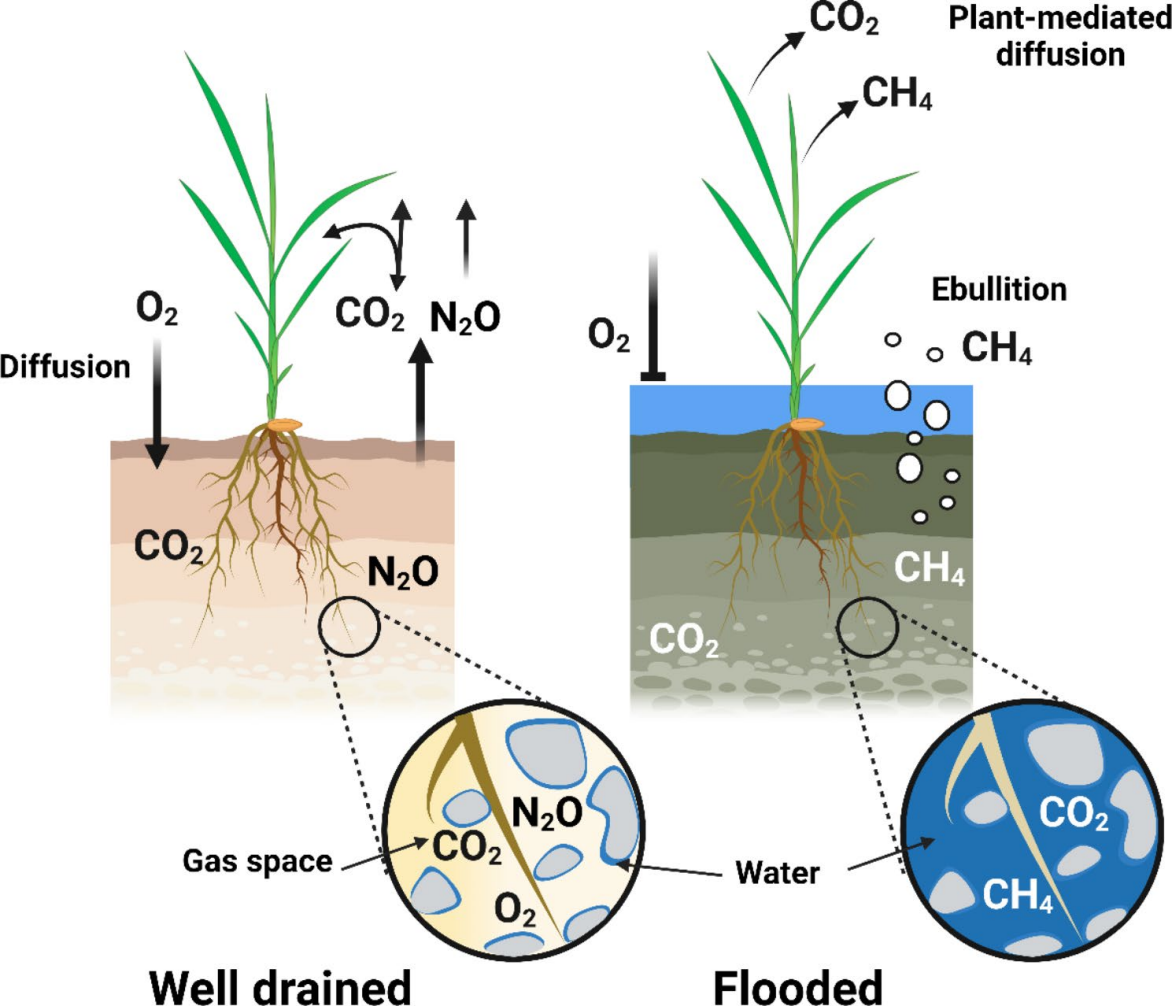
Overview of main greenhouse gases produced, and the pathways of emission from cultivated rice in a well-drained soil (left) or flooded paddy soil (right). In drained conditions, the porous spaces in the soils are gas-filled and contain molecular O_2_. CO_2_ and N_2_O are produced as a by-products of respiration, nitrification and denitrification. These gasses can easily diffuse from the soil to the atmosphere via the gas-filled pores. In flooded soils however, the porous spaces are water-filled and molecular diffusion of gases is highly impeded. CO_2_ and CH_4_ produced in respiration by roots or microorganism and in methanogensis accumulate to high concentration due to the slow diffusion. These greenhouse gases are primarily vented to the atmosphere through plant-mediated diffusion or to a lesser extent via ebullition. Created with BioRender.com.

**Fig. 2:**
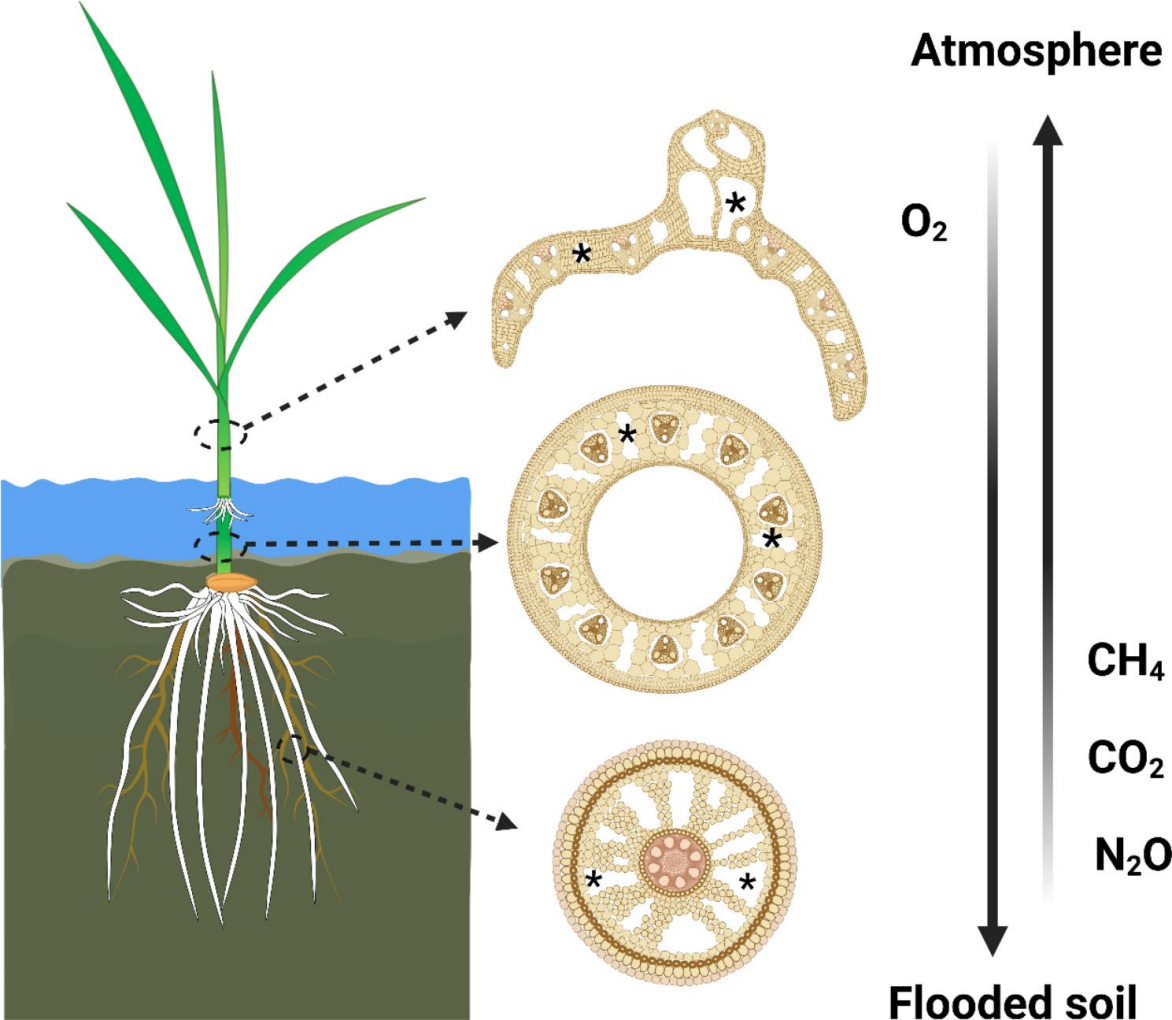
Plant-mediated diffusion of greenhouse gases from flooded soils to the atmosphere. Rice develop aerenchyma spaces in roots, internodes, and leaf blades, forming a low-resistance pathway (continuum of gas spaces), that facilitates the bidirectional diffusion of O_2_ from the atmosphere down to the flooded soil, and CH_4_, CO_2_ and/or N_2_O in the opposite direction, from the rhizosphere to the atmosphere. Asterisk keys in cross-sections from roots, internodes, and leaf blade tissues, indicate aerenchyma spaces. Created with BioRender.com.

Rice is the most important staple food, but current cultivation practices promote the formation and emission of greenhouse gases (Fig. 1). Carbon dioxide (CO_2_) and nitrous oxide (N_2_O) can accumulate in upland, drained soils, planted with rice, as a by-product of respiration by roots and microorganism, and nitrification and denitrification processes. These gasses can diffuse to the atmosphere via porous spaces in the soil. In lowland flooded soils, however, the porous spaces in soils are filled with water and molecular diffusion of gases is highly impeded (diffusion coefficient of gases in air is 10,000-fold faster than in water; Armstrong 1979). Therefore, CO_2_ and methane (CH_4_) accumulate to high concentrations as a by-product of respiration by roots and microorganisms, and production of CH_4_ by anaerobic methane-producing bacteria. In flooded soils, CO_2_ and CH_4_ are mainly emitted to the atmosphere through plant-mediated diffusion (c. 90%) or via ebullition of gas bubbles from the soil (c. 10%) (Figs. 1 and 2; Holzapfel-Pschorn et al. 1986; Schütz et al. 1989; Butterbach-Bahl and Rennenberg, 1997). Current rice cultivation accounts for 8% of the global anthropogenic CH_4_ emissions (Saunois et al. 2020), and 10% of global agriculturally related cropland N_2_O emissions (Wang et al. 2019), and therefore the potential to reduce greenhouse gas emissions from rice production systems is huge. We propose a strategy to reduce the carbon footprint from rice production by targetting breeding for roots traits that *i*) restrict the flux of CH_4_ and N_2_O to the shoot to reduce plant-mediated diffusion and *ii*) enhance the O_2_ flux to the flooded soil to facilitate aerobic mineralization (with production of CO_2_ instead of CH_4_) and increased CH_4_ oxidation. These root traits are the focus of the present review.

Management practices, climatic conditions, soil properties and plant characteristics directly influence the production rate of CH_4_ and N_2_O in rice paddies. Interestingly, production of CH_4_ and N_2_O in the soil is not facilitated by the same parameters. For example, CH_4_ production is reduced by draining flooded soils, but is increased by application of organic matter (mostly biochar), by N fertilization, in soils with a low bulk density and by rainfall during the growing season (Bo et al. 2022). In contrast, N_2_O production in a paddy soil is stimulated by drainage of the flooded soil, N fertilization and high soil bulk density (Bo et al. 2022), making it difficult to formulate a management plan aiming at reducing the net warming potential of paddy rice. Nevertheless, it is generally agreed that management schemes aiming at reducing water use also reduce greenhouse gas emissions from rice production systems. Discontinuous flooding schemes including direct seeding, alternative wetting and drying, intermittent irrigation and midseason drainage effectively reduce CH_4_ emissions with only few or no penalty on yield compared with continuous flooding conditions (Belder et al. 2004; Linquist et al. 2015). In fact, CH_4_ emissions have been predicted to decline by 54% using discontinuous flooding schemes, whereas N_2_O emissions almost double, but the net warming potential obtained by using discontinuous flooding schemes will still represent a reduction in 56% (Bo et al. 2022). Based on the above, changes in management practices have a large potential to reduce greenhouse gas emissions from rice production systems. However, in order to accelerate the reduction in emissions, we suggest to also focus on root traits that either enhance oxidation to prevent formation of CH_4_ and N_2_O, or reduce the penetration of these gases into the root system with subsequent plant-mediated diffusion via the root-shoot continuum of gas spaces (see section on aerenchyma below and Fig. 2).

**Fig. 3:**
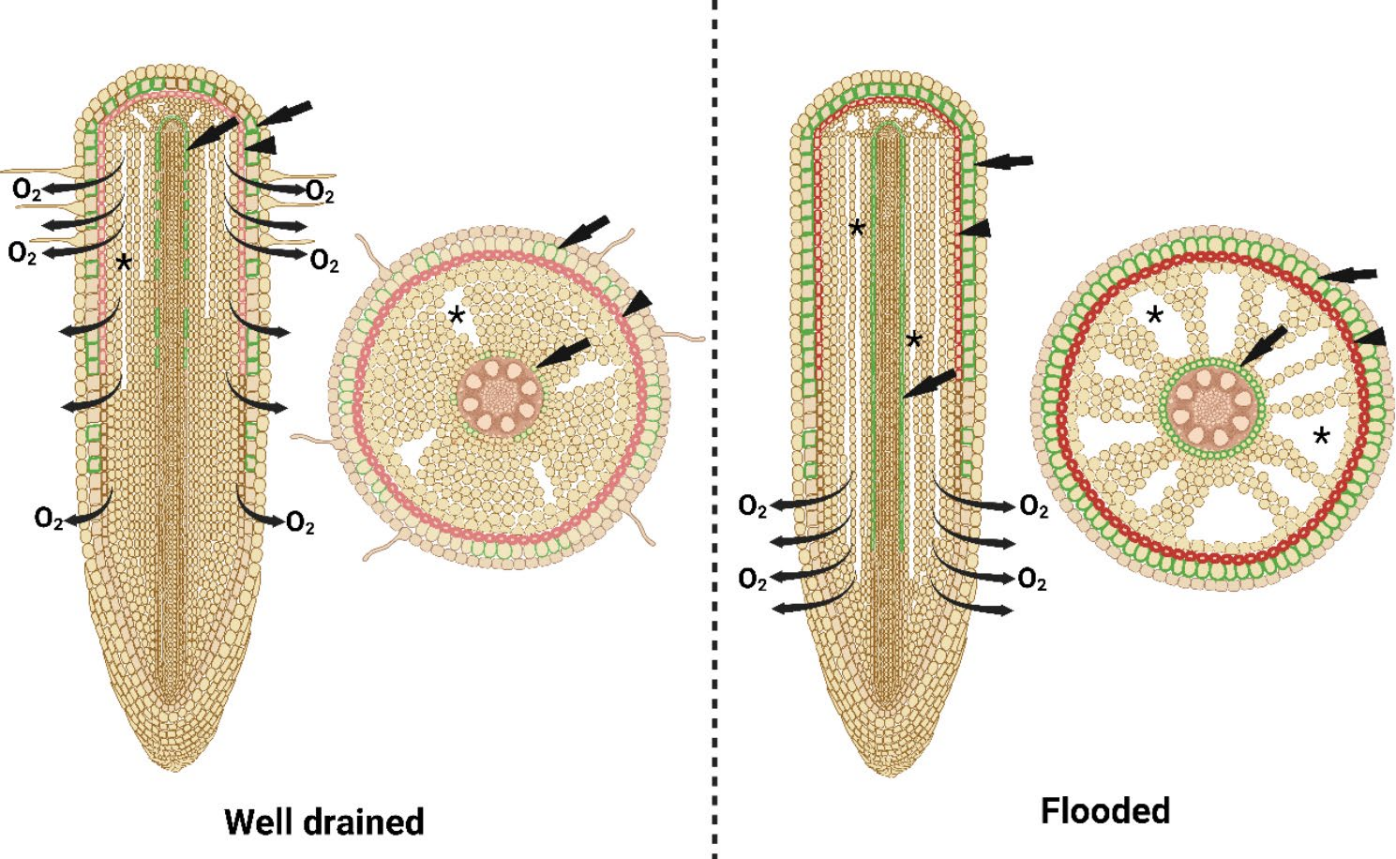
Anatomical characteristics of adventitious roots of rice grown in a well-drained soil (left) or in a flooded soil (right). Rice roots develop constitutive aerenchyma and its amount increases significantly when growing in a flooded soil. Suberization of exodermal and lignification of sclerenchyma cell walls are incipient in adventitious roots of rice growing in well-drained conditions, but the deposition of these polymers significantly increases when plants grow in flooded soils, forming complete suberized and lignified lamellae. These two polymers act as an apoplastic barrier impeding radial O_2_ loss. In roots growing in well-drained conditions, barriers to radial O_2_ loss are not constitutively formed, therefore, radial diffusion of O_2_ occurs along the root. In contrast, roots growing in flooded soils develop tight barriers to radial O_2_ loss in their basal parts, thus O_2_ effectively diffuses towards the root tip where O_2_ leaks into the rhizosphere since a barrier is not formed in this part of the root. Asterisk keys indicate aerenchyma spaces; arrowheads and arrows point to lignified and suberized cells, respectively. Created with BioRender.com

Rice is well-adapted to flooded soils. When growing in flooded soils, rice develops new adventitious roots with increased aerenchyma as well as barriers in the outer part of the root to impede radial O_2_ loss (ROL; see section below) in the basal parts of the roots (Fig. 3). These characteristics enable an effective internal aeration system allowing O_2_ diffusion from shoot to the root tip and therefore facilitating root respiration (Armstrong 1979), soil oxidation (Revsbech et al. 1999) and methanotrophy (Damgaard and Revsbech 1997). In contrast, flooding-sensitive plants, unable to maintain root growth and survive prolonged floodings, are characterised by having roots with only little aerenchyma and a weak, or absent, barrier to ROL (i.e., wheat, McDonald et al., 2002; maize, Abiko et al., 2012; *Urochloa* spp, Jiménez et al. 2021a). Consequently, high root porosity in combination with a tight barrier to ROL in the basal parts of the roots are considered the most important root adaptations allowing root grow in flooded, anoxic soils (Pedersen et al. 2021a).

**Fig. 4:**
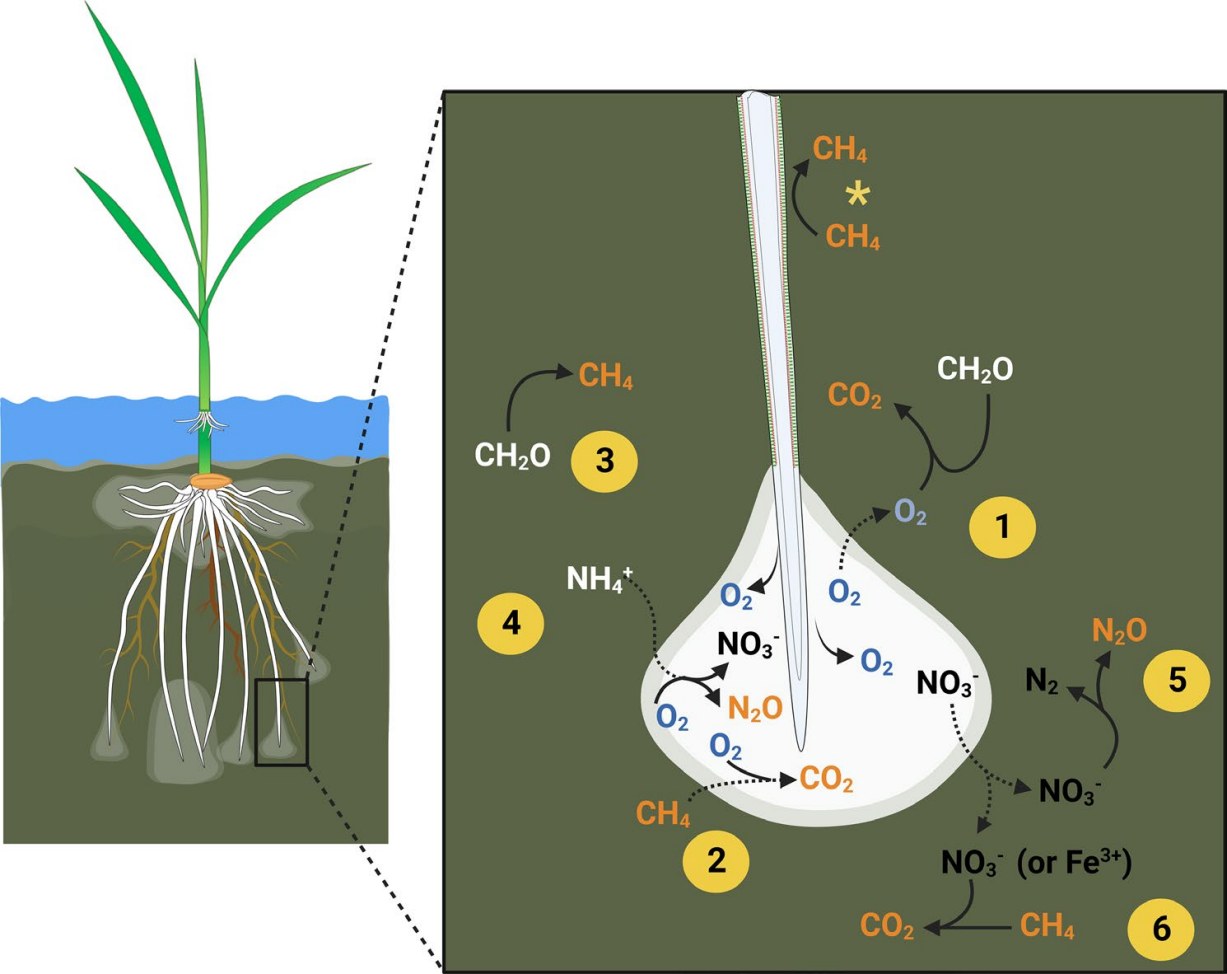
Young rice plant growing in a paddy field with flooded soil (left) and details of the biogeochemical reactions in the rhizosphere (right). Left: formation of new adventitious roots with enhanced aerenchyma spaces and tight barriers to radial O_2_ loss enables deep rooting in the flooded soil resulting in rhizosphere oxidation, especially in regions near the root tip where the barriers to radial O_2_ loss are rarely formed (see Fig. 3). Right: oxic zones in the interface between roots and rhizosphere facilitate aerobic mineralization with formation of CO_2_ (1), CH_4_ oxidation to CO_2_ (2) but still with CH_4_ production in the surrounding anoxic soil (3). The oxic zones also support nitrification (conversion of NH_4_^+^ into NO_3_^−^) (4) with some of the NO_3_^−^ feeding into denitrification (5) or aerobic methanotrophy (6). The barrier to radial O_2_ loss formed on the basal parts of the roots restricts inward radial diffusion of CH_4_ (*) preventing plant-mediated diffusion of CH_4_ (Fig. 2). Diffusion in or out of the oxic zones are indicated by stippled lines, and greenhouse gases are indicated in orange colour font. Created with BioRender.com.

In this review, we focus on the root morphological and anatomical changes of rice roots growing in flooded soils, which can be exploited to reduce the production and plant-mediated diffusion of greenhouse gases from paddy fields. We propose an innovative approach, through enhanced soil oxidation and reduced gas diffusion from soils into roots (Fig. 4), to reduce greenhouse gas emissions. Our focus is on greenhouse gas emissions via plant-mediated diffusion rather than passive diffusion or ebullition from soils as the former accounts for the vast majority of total emissions (Holzapfel-Pschorn et al. 1986; Schütz et al. 1989; Butterbach-Bahl and Rennenberg, 1997). The wider effect of rhizosphere oxidation by O_2_ released from roots and its effect on reduced metals and formation of metal plaques is not emphasized in depth (see Ponnamperuma 1972; Kirk 2004). Likewise, emphasis is given to an eco-physiological approach and not genetic regulations of such root traits as this has been the focus of previous reviews (Rebouillat et al. 2008; Meng et al. 2019).

## The Root System

Worldwide, 75% of rice production occurs in flooded soils (Bouman et al., 2007). Upon flooding, the gases in the pore spaces of the soil are replaced by water, and O_2_ is rapidly consumed by roots and aerobic microorganisms, limiting root respiration, growth and nutrient uptake of flood-sensitive plants (Colmer et al. 2014; Kirk et al. 2014). Root anatomical modifications enabling effective internal O_2_ diffusion from above-water tissues to the submerged roots sustain rice growth and production in flooded soils (Colmer et al. 1998; Colmer 2003a, b; Figs. 2 and 3). Anatomical, eco-physiological and genetic studies underpin our current understanding on how rice responds and adapts to flooded soils, and these studies have been pivotal for developing rice cultivars that are better adapted to flooded soils or even complete submergence (Mackill et al. 2012). Moreover, the evaluation and characterization of undomesticated wild rice species (i.e., Ejiri and Shiono 2020; Tong et al. 2023) increases our chances to breed high-yielding elite cultivars with desirable characteristics and reduced environmental footprint, including a reduction in greenhouse gas emissions.

Rice plants display a characteristic monocotyledonous fibrous root system containing seminal, adventitious (crown) and lateral roots (Freschet et al. 2021). The seminal root originates from the embryo allowing initial nutrient uptake and anchorage, but the root system is later dominated by adventitious roots emerging from nodes on the stem and tillers (Rebouillat et al. 2008). Both seminal and adventitious roots develop secondary lateral roots. These lateral roots are classified into short-type (S-type) or long-type (L-Type; Yamauchi et al. 1987). Seminal and adventitious roots are usually longer and thicker than lateral roots, but due to the high number of lateral roots, their contribution to total root lengths and root external surface is often higher than that of seminal and adventitious roots (Kirk 2003).

Root architecture is altered when rice grow in flooded soils compared with a drained soil. The emergence of adventitious roots is highly stimulated upon soil flooding (Mergemann and Sauter 2000; Colmer 2003a). Numerous adventitious roots (either formed below soil surface or as aquatic adventitious root developed from nodes above the soil surface) functionally replace seminal and old adventitious roots, which are no longer capable of maintaining root growth and nutrient uptake due to insufficient respiratory O_2_ supply in the anoxic soil. New adventitious roots formed after flooding are usually devoid of S-type lateral roots, and display bigger diameters and higher aerenchyma spaces (see section below) in comparison with roots formed prior to flooding. The new adventitious roots enable anchorage and sustain nutrient uptake (Zhang et al. 2017), and in the case of aquatic adventitious roots they also take up O_2_ from the floodwater (Ayi et al. 2016; Lin et al. 2021). Evaluating a set of different rice types (i.e., paddy, upland, and deep-water rice), it was found that the number of adventitious roots per plant growing in flooded soils increased 1.1-2.5-fold in comparison with their respective drained controls (Colmer 2003a). New adventitious roots can develop L-type lateral roots, and these can further develop S-type lateral roots. Therefore, the formation of thick, aerenchymous adventitious roots with barriers to impede radial O_2_ loss to the soil (see section below) allowing O_2_ diffusion to both the apical parts and the lateral roots represent the best compromise for growth and nutrient uptake in paddy fields (Kirk 2003).

Despite an increased number of adventitious roots formed in flooded anoxic soils, these roots are usually shorter than the ones formed in a drained soil (Colmer 2003a; Colmer et al. 2006; Kotula et al. 2009). The slower growth rate is mainly owing to O_2_ deficiency as the tip grow further from the O_2_ source (Armstrong 1979), although chemically reduced compounds produced in flooded soils can also impair root growth (e.g., H_2_S, Armstrong and Armstrong 2005; organic acids, Colmer et al. 2019). Root penetration into a flooded soil is determined by its internal capacity to diffuse O_2_ from the shoot to the root tips; the root extension of rice is reduced or ceases completely below a critical O_2_ partial pressure ≤ 0.8 kPa (Armstrong and Webb 1985). Rice genotypes with higher capacity for internal O_2_ diffusion can grow faster and produce longer roots in flooded soils (Colmer 2003a). An effective internal O_2_ diffusion system has also a significant influence on lateral root development, as lateral roots rely on the O_2_ available from the aerenchyma within the main axis. Newly developed adventitious roots and laterals in flooded soils are major sources of O_2_ loss (cf. Kirk 2003), contributing to CH_4_ oxidation. Hence, the development of large oxic zones in the interface between roots and bulk soil will also favour nitrification of the NH_4_^+^ diffusing in from the neighbouring anoxic soil (Kirk 2004). As the soil oxygenation increases proportionally to the number of roots produced, rice genotypes with bigger root systems (i.e., longer roots, higher root dry mass and volume) and/or increased porosity have been associated with low CH_4_ emissions (Jiang et al. 2017; Chen et al. 2019; Ding et al. 2021). A bigger root system in flooded soils will allow for higher resource exploitation and rhizosphere oxidation but will also increase the CO_2_ production due to higher root respiratory activities.

High variation in root angles exist within rice genotypes (Lafitte et al. 2001; Uga et al. 2012). Provided that root growth into a flooded soil solely rely on internal O_2_ diffusion from the shoot, the potential benefits for a specific root phenotype with shallow or steep root angles is not obvious. Interestingly, rice genotypes with steep angles (deeper roots) are better adapted to droughts (Uga et al. 2013) compared with genotypes with shallow angles (superficial roots), but the latter genotypes have shown better performance during soil compaction (Ramalingam et al. 2017) or when exposed to salinity stress (Kitomi et al. 2020). Development of superficial roots near the soil surface could benefit from layers with higher O_2_ availability (Pedersen et al. 2021a), however, higher oxidation of chemically reduced compounds could also slow growth as some nutrients (e.g., Fe and P) are less available in oxidized soils. Genotypes with steeper root angles resulting in deep rooting and enhanced diffusion of O_2_ down to the deep anoxic layers in the soil should therefore be targeted to increase CH_4_ oxidation and thereby reduce emissions.

## Root Aerenchyma

Aerenchyma starts forming in the cortex when the roots grow in flooded soils. Aerenchyma in rice roots is enlarged gas-filled spaces formed via programmed cell death that facilitates diffusion of gases (Yamauchi et al. 2019a). In rice, aerenchyma tissues develop in roots, internodes, sheaths, leaf lamina midrib and leaf blades (Matsukura et al. 2000; Colmer and Pedersen 2008; Steffens et al. 2011), forming a continuum of gas spaces that accelerates diffusion of all gases (e.g., O_2_, CO_2_, CH_4_, N_2_O and ethylene) between the rhizosphere and the atmosphere above (Fig. 2). Aerenchyma is constitutively formed in the cortex of rice roots, but its proportion significantly increases when roots are formed in flooded soils due to formation of inducible aerenchyma (Colmer et al., 1998; 2003a; Yamauchi and Nakazono 2022). Total root porosity (including aerenchyma and small intercellular spaces) of adventitious roots of different rice genotypes increased from 12 to 38% in aerated conditions to 22–48% when growing in anoxic substrates (Colmer et al. 1998; McDonald et al. 2002; Colmer 2003a; Tong et al. 2023). In adventitious roots of rice, porosity is higher in the basal parts near the root-shoot junction and declines towards the root tip (porosity was *c*. 34% or 43–54% at 60 mm behind root tip but decreased to *c*. 0% or 0–9% at 10 mm behind the root tip for roots growing in aerated or anoxic conditions, respectively, Kotula et al. 2009; Yamauchi et al. 2019b). Similarly, aerenchyma declined from 12 to 32% at 50 mm behind the root tip to 8–14% at 20 mm behind the root tip for L-type lateral roots developed in aerated or anoxic conditions, respectively (Noorrohmah et al. 2020). Interestingly, aerenchyma does not form in S-type lateral roots (Noorrohmah et al. 2020). Importantly, although increased aerenchyma formation allows for higher O_2_ diffusion from shoot to roots, greenhouse gases produced in flooded soils can diffuse via this low-resistance pathway in the opposite direction, i.e., from flooded soils to the atmosphere (Henneberg et al. 2012; Kirk et al. 2019; Fig. 2).

Compared with the widely studied O_2_ diffusion through aerenchyma spaces, little is known about diffusion of greenhouse gases through plant tissues. Oxygen can diffuse longitudinally (axially) and radially through and across the roots (Armstrong and Beckett 1987). Internal O_2_ diffusion from the shoot and down to roots in flooded soils is determined by root anatomical and physiological characteristics including root porosity, root length, stele and cortex diameters, cellular respiration, and cell wall composition (Armstrong et al. 1983; Armstrong and Beckett 1987; Colmer 2003b, Pedersen et al. 2021a; Jiménez et al. 2021a). However, the diffusion of other gases (including CO_2_, CH_4_ and N_2_O) from the rhizosphere into and along the roots, and the root characteristics influencing such gas diffusion processes, remain largely unknown, although some attempts to understand this process have been done for CH_4_ (Nouchi et al. 1990; Beckett et al. 2001). Most experiments published succeed to quantify fluxes of greenhouse gases emitted from soils or plant tissues under specific circumstances but fail to relate these fluxes with the anatomical and/or physiological root characteristics governing internal gas diffusion. Significant variation in root aerenchyma development among rice genotypes exists (Colmer 2003a), and the identification and characterization of wild rice species with contrasting root anatomical characteristics (Tong et al. 2023) pave the way for studies on the influence of root traits in the production, oxidation and/or emission of greenhouse gases from flooded paddy soils.

## Root Apoplastic Barriers

Rice can form barriers in the outer parts of the roots to impede radial O_2_ loss to the flooded, anoxic rhizosphere (Colmer 2003a). These barriers enable an effective longitudinal O_2_ diffusion from the basal parts of roots to the growing tips (Fig. 3). The formation of barriers to radial O_2_ loss coincide with increased impregnation of suberin and lignin in the cell walls of the exodermis and sclerenchyma, respectively (Kotula et al. 2009; Ranathunge et al. 2011). In addition to restrict the O_2_ loss from root to rhizosphere, the barriers can also reduce the entry of gases in the opposite direction, i.e., from rhizosphere and into the root (shown for H_2_, Peralta Ogorek et al. 2021and H_2_S, Peralta Ogorek et al., 2023). The barrier to radial O_2_ loss in adventitious roots of rice is induced by different environmental cues including reduced Fe (Mongon et al. 2014), sulfides (Armstrong and Armstrong 2005) and low molecular mass organic acids (Colmer et al. 2019). However, the barrier is not formed in roots in aerated conditions (except for some wild rice species that constitutively form a barrier to radial O_2_ loss; Ejiri et al. 2020). Higher basipetal radial O_2_ loss rates decreasing towards the root tip are characteristic patterns of roots with no or only a weak barrier to radial O_2_ loss, while lower rates of radial O_2_ loss along basal zones but increasing towards the highly permeable root tip, indicate the presence of a tight barrier (see Fig. 1 in Jiménez et al. 2021b for characteristic radial O_2_ loss patterns from roots of rice with or without barriers to radial O_2_ loss). There is significant phenotypic variation in the ´tightness` (i.e., permeance to O_2_ through exodermal/hypodermal cell layers) of the barrier to radial O_2_ loss in adventitious roots of rice (Colmer et al., 1988; Colmer 2003a; Tong et al. 2023). Moreover, the barrier to radial O_2_ loss can also be formed in L-type lateral roots while these never form in the S-type (Noorrohmah et al. 2020). Radial O_2_ loss into anoxic soils often results in characteristic plaque formation on the root surfaces. Root plaques derive from oxidation of reduced metals such as Mn^2+^ and Fe^2+^ resulting in metal oxides such as ferrihydrite and goethite that can completely cover the root epidermis forming a sheath (Hansel et al. 2001). These plaques can enhance nutrient uptake (Jiang et al. 2009), but they can also serve a role as an alternative barrier to radial O_2_ loss in roots of wetland plants that are unable to deposit suberin or lignin in the outer cell walls (Møller and Sand-Jensen 2008).

The formation of barriers to impede radial O_2_ loss enable root growth into flooded soils. Mathematical modelling computing root anatomical characteristics indicated that the maximum root length attained in flooded soils may increase from 230 to 590 mm in adventitious roots of rice without or with barriers to radial O_2_ loss, respectively (Pedersen et al., 2021a). Moreover, modelling showed that lateral roots of *Zea nicaraguensis* can grow to a maximum length of 74 mm with a barrier, but only to 33 mm without a barrier to radial O_2_ loss (Pedersen et al., 2021b). An extensive root system will translate into bigger zones with appropriate conditions for CH_4_ oxidation, particularly close to the highly permeable root tips. Moreover, a considerable O_2_ loss can come from newly formed, short adventitious roots (Colmer et al. 2006; Shiono et al. 2011; Ejiri et al. 2021) and lateral roots of rice (Noorrohmah et al. 2020) that do not develop barriers to radial O_2_ loss. In addition, the occurrence of ´windows´ without suberin or lignin impregnation in the cell walls near the sites for lateral root emergence in adventitious roots can potentially be active sites for radial O_2_ loss (Armstrong et al., 2000; Soukup et al. 2002; Ejiri and Shiono 2020; Jiménez et al. 2021c). However, increased radial O_2_ loss from several sites along roots, or in specific types of roots, can come with drawbacks as these hotspots can also be sites of increased inward diffusion of greenhouse gases from the flooded soil. Interestingly, no study has so far addressed these *pros* and *cons* of diffusional O_2_ loss to the flooded soils and the possible impact on plant-mediated diffusion of CO_2_, CH_4_ or N_2_O with subsequent emission into the atmosphere.

## Potential for Mitigation of Greenhouse Gases via Manipulation of Key Root Traits in Rice

Increased aerenchyma together with the formation of barriers to impede radial O_2_ loss in basal parts of the roots act synergistically to enhance internal O_2_ diffusion (Armstrong 1979; Colmer 2003b; Fig. 3). The exploitation of these traits appears as a solution to reduce the production, plant-mediated diffusion, and emission of greenhouse gases from flooded rice paddies. Roots of rice with increased aerenchyma formation and tight barriers to radial O_2_ loss releasing O_2_ to the deep layers of the submerged soils will enable higher rhizosphere CH_4_ oxidation and nitrification of NH_4_^+^; while the entry of greenhouse gases into the basal parts of the root is limited given the very low permeability of the root cell walls to gases (Fig. 4). We therefore propose that screening for rice genotypes with high aerenchyma formation and tight barriers to radial O_2_ loss by breeding programs is a promising way to increase the soil oxidizing power of new rice varieties.

Oxygen loss from roots to flooded, anoxic soils promote a favourable habitat for CH_4 _oxidizing microorganisms (Jiang et al. 2017; Chen et al. 2019) but can also favour conditions for enhanced coupled nitrification-denitrification (Risgaard-Petersen and Jensen 1997; Kirk 2004; Kirk and Kronzucker 2005). Modern rice genotypes must therefore display specific root traits that can provide a soil redox potential in a range that maintains both N_2_O and CH_4_ emissions low (e.g., + 200 to -100 mV, Hou et al. 2000). Interestingly, the oxic zones near the root tips of rice roots has also been shown to display characteristic diurnal patterns with spatial expansion during the daytime due to net O_2_ production in photosynthesis and shrinkage during darkness due to net O_2_ consumption (Larsen et al. 2015). To our knowledge, it has not yet been studied in detail how such spatial fluctuations in molecular O_2_ influence the production of, e.g., N_2_O, but it is known that sudden restrictions in O_2_ availability promotes N_2_O production both during nitrification and denitrification (Wrage et al. 2001).

Some efforts have been done to understand the influence of rice roots characteristics on greenhouse gases emissions from flooded soils (e.g., root aerenchyma and CO_2_ venting, Kirk et al. 2019; total root volume and root areas on CH_4_ emissions, Ding et al. 2021), but several aspects limit our capacity to produce elite rice cultivars with a reduced carbon footprint. To date, these limitations include: *i*) lacking information on the permeability of roots to CO_2_, CH_4_ and N_2_O, *ii*) lack of knowledge on greenhouse gases dynamics within aerobic roots, *iii*) lacking knowledge on spatial and temporal in-vivo dynamics for greenhouse gases in oxidized or reduced areas of soils, and *iv*) lack of understanding of the influence of root anatomical as well as root chemical characteristics on diffusion of greenhouse gases from soils and throughout and along roots.

## Conclusions

Considering the existent phenotypic variability in root traits of rice genotypes, we propose that the development of new rice cultivars exhibiting an effective internal O_2_ diffusion system, through enhanced aerenchyma formation and development of tight barriers to impede radial O_2_ loss along the basal parts of the roots will increase CH_4_ oxidation as well as promote nitrification, reducing the production of greenhouse gases and benefitting plant nutrient uptake. Moreover, the development of roots with tight barriers to impede radial O_2_ loss, would also limit the radial diffusional entry of greenhouse gases into roots and further reduce the plant-mediated diffusion of these gases from paddy fields.

## Data Availability

Not applicable.

## References

[CR600] Abiko, T., Kotula, L., Shiono, K., Malik, A. I., Colmer, T. D., & Nakazono, M. (2012). Enhanced formation of aerenchymaand induction of a barrier to radial oxygen loss in adventitious roots of Zea nicaraguensis contribute to itswaterlogging tolerance as compared with maize (Zea mays ssp. mays). *Plant Cell Environ*, *35*(9), 1618–1630. 10.1111/j.1365-3040.2012.02513.x 10.1111/j.1365-3040.2012.02513.x 22471697

[CR1] Armstrong W (1979) Aeration in higher plants, vol 7. Academic Press

[CR6] Armstrong W (2000). Oxygen distribution in Wetland Plant roots and permeability barriers to gas-exchange with the Rhizosphere: a Microelectrode and Modelling Study with Phragmites australis. Ann Botany.

[CR5] Armstrong J, Armstrong W (2005). Rice: sulfide-induced barriers to root radial oxygen loss, Fe2 + and water uptake, and lateral root emergence. Ann Bot.

[CR4] Armstrong W, Beckett PM (1987). Internal aeration and the development of Stelar Anoxia in Submerged roots. A Multishelled Mathematical Model combining Axial Diffusion of Oxygen in the cortex with radial losses to the stele, the Wall Layers and the Rhizosphere. New Phytol.

[CR3] Armstrong W, Webb T (1985). A critical oxygen pressure for root extension in rice. J Exp Bot.

[CR2] Armstrong W, Healy MT, Lythe S (1983). Oxygen diffusion in pea ii. Oxygen concentrations in the primary pea root apex as affected by growth, the production of laterals and radial oxygen loss. New Phytol.

[CR7] Ayi Q, Zeng B, Liu J, Li S, van Bodegom PM, Cornelissen JHC (2016). Oxygen absorption by adventitious roots promotes the survival of completely submerged terrestrial plants. Ann Bot.

[CR8] Beckett PM, Armstrong W, Armstrong J (2001). Mathematical modelling of methane transport by Phragmites: the potential for diffusion within the roots and rhizosphere. Aquat Bot.

[CR9] Belder P, Bouman B, Cabangon R, Guoan L, Quilang E, Yuanhua L, Spiertz J, Tuong T (2004). Effect of water-saving irrigation on rice yield and water use in typical lowland conditions in Asia. Agric Water Manage.

[CR10] Bo Y, Jägermeyr J, Yin Z, Jiang Y, Xu J, Liang H, Zhou F (2022). Global benefits of non-continuous flooding to reduce greenhouse gases and irrigation water use without rice yield penalty. Glob Change Biol.

[CR11] Bouman BAM, Tuong LRM (2007). Water management in irrigated rice.

[CR12] Butterbach-Bahl K, Papen H, Rennenberg H (1997).

[CR13] Chen Y, Li S, Zhang Y, Li T, Ge H, Xia S, Gu J, Zhang H, Lü B, Wu X, Wang Z, Yang J, Zhang J, Liu L (2019). Rice root morphological and physiological traits interaction with rhizosphere soil and its effect on methane emissions in paddy fields. Soil Biol Biochem.

[CR15] Colmer TD (2003a) Aerenchyma and an inducible barrier to radial oxygen loss facilitate root aeration in upland, paddy and deep-water rice (Oryza sativa L). Ann Bot 91 Spec No 2301–309. 10.1093/aob/mcf114 10.1093/aob/mcf114 PMC4795684 12509350

[CR16] Colmer TD (2003). Long-distance transport of gases in plants: a perspective on internal aeration and radial oxygen loss from roots. Plant Cell Environ.

[CR18] Colmer TD, Pedersen O (2008). Oxygen dynamics in submerged rice (Oryza sativa). New Phytol.

[CR14] Colmer TD, Gibberd MR, Wiengweera A, Tinh TK (1998). The barrier to radial oxygen loss from roots of rice (Oryza sativa L.) is induced by growth in stagnant solution. J Exp Bot.

[CR17] Colmer TD, Cox MC, Voesenek LA (2006). Root aeration in rice (Oryza sativa): evaluation of oxygen, carbon dioxide, and ethylene as possible regulators of root acclimatizations. New Phytol.

[CR19] Colmer TD, Armstrong W, Greenway H, Ismail AM, Kirk GJD, Atwell BJ (2014) Physiological Mechanisms of Flooding Tolerance in Rice: Transient Complete Submergence and Prolonged Standing Water. In Progress in Botany (pp. 255–307). 10.1007/978-3-642-38797-5_9

[CR20] Colmer TD, Kotula L, Malik AI, Takahashi H, Konnerup D, Nakazono M, Pedersen O (2019). Rice acclimation to soil flooding: low concentrations of organic acids can trigger a barrier to radial oxygen loss in roots. Plant Cell Environ.

[CR21] Damgaard LR, Revsbech NP (1997). A microscale biosensor for methane containing methanotrophic bacteria and an internal oxygen reservoir. Anal Chem.

[CR22] Ding H, Jiang Y, Cao C (2021). Deep rice root systems reduce methane emissions in rice paddies. Plant Soil.

[CR24] Ejiri M, Shiono K (2020). Groups of multi-cellular passage cells in the root exodermis of Echinochloa crus-galli varieties lack not only suberin lamellae but also lignin deposits. Plant Signal Behav.

[CR23] Ejiri M, Sawazaki Y, Shiono K (2020) Some accessions of amazonian Wild Rice (Oryza glumaepatula) Constitutively Form a barrier to Radial Oxygen loss along Adventitious roots under aerated conditions. Plants (Basel) 9(7). 10.3390/plants9070880 10.3390/plants9070880 PMC7412225 32668711

[CR25] Ejiri M, Fukao T, Miyashita T, Shiono K (2021). A barrier to radial oxygen loss helps the root system cope with waterlogging-induced hypoxia. Breed Sci.

[CR26] Freschet GT, Pages L, Iversen CM, Comas LH, Rewald B, Roumet C, Klimesova J, Zadworny M, Poorter H, Postma JA, Adams TS, Bagniewska-Zadworna A, Bengough AG, Blancaflor EB, Brunner I, Cornelissen JHC, Garnier E, Gessler A, Hobbie SE, Meier IC, Mommer L, Picon-Cochard C, Rose L, Ryser P, Scherer-Lorenzen M, Soudzilovskaia NA, Stokes A, Sun T, Valverde-Barrantes OJ, Weemstra M, Weigelt A, Wurzburger N, York LM, Batterman SA, Gomes, de Moraes M, Janecek S, Lambers H, Salmon V, Tharayil N, McCormack ML (2021) A starting guide to root ecology: strengthening ecological concepts and standardising root classification, sampling, processing and trait measurements. New Phytol, 232(3), 973–1122. 10.1111/nph.17572 10.1111/nph.17572 PMC8518129 34608637

[CR27] Hansel CM, Fendorf S, Sutton S, Newville M (2001). Characterization of Fe plaque and associated metals on the roots of mine-waste impacted aquatic plants. Environ Sci Technol.

[CR29] Henneberg A, Sorrell BK, Brix H (2012). Internal methane transport through Juncus effusus: experimental manipulation of morphological barriers to test above- and below-ground diffusion limitation. New Phytol.

[CR28] Holzapfel-Pschorn A, Conrad R, Seiler W (1986). Effects of vegetation on the emission of methane from submerged paddy soil. Plant Soil.

[CR30] Hou AX, Chen GX, Wang ZP, Van Cleemput O (2000). Methane and nitrous oxide emissions from a rice field in relation to soil redox and microbiological processes. Soil Sci Soc Am J.

[CR31] Jiang FY, Chen X, Luo AC (2009). Iron plaque formation on wetland plants and its influence on phosphorus, calcium and metal uptake. Aquat Ecol.

[CR32] Jiang Y, van Groenigen KJ, Huang S, Hungate BA, van Kessel C, Hu S, Zhang J, Wu L, Yan X, Wang L, Chen J, Hang X, Zhang Y, Horwath WR, Ye R, Linquist BA, Song Z, Zheng C, Deng A, Zhang W (2017). Higher yields and lower methane emissions with new rice cultivars. Glob Chang Biol.

[CR33] Jiménez JdlC, Cardoso JA, Kotula L, Veneklaas EJ, Pedersen O, Colmer TD (2021). Root length is proxy for high-throughput screening of waterlogging tolerance in Urochloa spp. grasses. Funct Plant Biol.

[CR34] Jiménez JdlC, Pellegrini E, Pedersen O, Nakazono M (2021b) Radial oxygen loss from plant roots-methods. Plants (Basel) 10(11). 10.3390/plants10112322 10.3390/plants10112322 PMC8622749 34834684

[CR35] Jiménez JdlC, Clode PL, Signorelli S, Veneklaas EJ, Colmer TD, Kotula L (2021). The barrier to radial oxygen loss impedes the apoplastic entry of iron into the roots of Urochloa humidicola. J Exp Bot.

[CR36] Kirk GJD (2003). Rice root properties for internal aeration and efficient nutrient acquisition in submerged soil. New Phytol.

[CR37] Kirk GJ (2004) Reduction and Oxidation. In The Biogeochemistry of Submerged Soils (pp. 93–134). 10.1002/047086303X.ch4

[CR38] Kirk GJ, Kronzucker HJ (2005). The potential for nitrification and nitrate uptake in the rhizosphere of wetland plants: a modelling study. Ann Bot.

[CR39] Kirk GJD, Greenway H, Atwell BJ, Ismail AM, Colmer TD (2014) Adaptation of Rice to Flooded Soils. In Progress in Botany (pp. 215–253). 10.1007/978-3-642-38797-5_8

[CR40] Kirk GJD, Boghi A, Affholder MC, Keyes SD, Heppell J, Roose T (2019). Soil carbon dioxide venting through rice roots. Plant Cell Environ.

[CR41] Kitomi Y, Hanzawa E, Kuya N, Inoue H, Hara N, Kawai S, Kanno N, Endo M, Sugimoto K, Yamazaki T, Sakamoto S, Sentoku N, Wu J, Kanno H, Mitsuda N, Toriyama K, Sato T, Uga Y (2020). Root angle modifications by the DRO1 homolog improve rice yields in saline paddy fields. Proc Natl Acad Sci U S A.

[CR42] Kotula L, Ranathunge K, Schreiber L, Steudle E (2009). Functional and chemical comparison of apoplastic barriers to radial oxygen loss in roots of rice (Oryza sativa L.) grown in aerated or deoxygenated solution. J Exp Bot.

[CR43] Lafitte HR, Champoux MC, McLaren G, O’Toole JC (2001). Rice root morphological traits are related to isozyme group and adaptation. Field Crops Research.

[CR44] Larsen M, Santner J, Oburger E, Wenzel WW, Glud RN (2015). O2 dynamics in the rhizosphere of young rice plants (Oryza sativa L.) as studied by planar optodes. Plant Soil.

[CR45] Lin C, Ogorek LLP, Pedersen O, Sauter M (2021). Oxygen in the air and oxygen dissolved in the floodwater both sustain growth of aquatic adventitious roots in rice. J Exp Bot.

[CR46] Linquist BA, Anders MM, Adviento-Borbe MA, Chaney RL, Nalley LL, da Rosa EF, van Kessel C (2015). Reducing greenhouse gas emissions, water use, and grain arsenic levels in rice systems. Glob Chang Biol.

[CR48] Mackill DJ, Ismail AM, Singh US, Labios RV, Paris TR (2012) Development and Rapid Adoption of Submergence-Tolerant (Sub1) Rice Varieties. In (pp. 299–352). 10.1016/b978-0-12-394276-0.00006-8

[CR49] Matsukura C, Kawai M, Toyofuku K, Barrero RA, Uchimiya H, Yamaguchi J (2000). Transverse vein differentiation associated with gas space formation—fate of the middle cell layer in leaf sheath development of rice. Ann Botany.

[CR47] McDonald MP, Galwey NW, Colmer TD (2002). Similarity and diversity in adventitious root anatomy as related to root aeration among a range of wetland and dryland grass species. Plant Cell Environ.

[CR50] Meng F, Xiang D, Zhu J, Li Y, Mao C (2019). Molecular Mechanisms of Root Development in Rice. Rice (N Y).

[CR51] Mergemann H, Sauter M (2000). Ethylene induces epidermal cell death at the site of Adventitious Root Emergence in Rice1. Plant Physiol.

[CR53] Møller CL, Sand-Jensen K (2008). Iron plaques improve the oxygen supply to root meristems of the freshwater plant, Lobelia dortmanna. New Phytol.

[CR52] Mongon J, Konnerup D, Colmer TD, Rerkasem B (2014). Responses of rice to Fe^2+^ in aerated and stagnant conditions: growth, root porosity and radial oxygen loss barrier. Funct Plant Biol.

[CR54] Noorrohmah S, Takahashi H, Nakazono M (2020). Formation of a barrier to radial oxygen loss in L-type lateral roots of rice. Plant Root.

[CR55] Nouchi I, Mariko S, Aoki K (1990). Mechanism of methane transport from the Rhizosphere to the atmosphere through Rice plants 1. Plant Physiol.

[CR56] Pedersen O, Sauter M, Colmer TD, Nakazono M (2021). Regulation of root adaptive anatomical and morphological traits during low soil oxygen. New Phytol.

[CR57] Pedersen O, Nakayama Y, Yasue H, Kurokawa Y, Takahashi H, Heidi Floytrup A, Omori F, Mano Y, Colmer D, Nakazono M (2021). Lateral roots, in addition to adventitious roots, form a barrier to radial oxygen loss in Zea nicaraguensis and a chromosome segment introgression line in maize. New Phytol.

[CR58] Peralta Ogorek LL, Pellegrini E, Pedersen O (2021). Novel functions of the root barrier to radial oxygen loss - radial diffusion resistance to H_2_ and water vapour. New Phytol.

[CR59] Peralta Ogorek LL, Takahashi H, Nakazono M, Pedersen O The barrier to radial oxygen loss protects roots against hydrogen sulphide intrusion and its toxic effect. New Phytologist, n/a(n/a). 10.1111/nph.18883 10.1111/nph.18883 36928886

[CR60] Ponnamperuma FN (1972) Chemistry of submerged soils. Advances in Agronomy, vol 24. Academic Press, Inc., pp 29–96

[CR61] Ramalingam P, Kamoshita A, Deshmukh V, Yaginuma S, Uga Y (2017). Association between root growth angle and root length density of a near-isogenic line of IR64 rice with DEEPER ROOTING 1 under different levels of soil compaction. Plant Prod Sci.

[CR62] Ranathunge K, Lin J, Steudle E, Schreiber L (2011). Stagnant deoxygenated growth enhances root suberization and lignifications, but differentially affects water and NaCl permeabilities in rice (Oryza sativa L.) roots. Plant Cell Environ.

[CR63] Rebouillat J, Dievart A, Verdeil JL, Escoute J, Giese G, Breitler JC, Gantet P, Espeout S, Guiderdoni E, Périn C (2008). Molecular Genetics of Rice Root Development. Rice.

[CR64] Revsbech NP, Pedersen O, Reichardt W, Briones A (1999). Microsensor analysis of oxygen and pH in the rice rhizosphere under field and laboratory conditions. Biol Fertil Soils.

[CR65] Risgaard-Petersen N, Jensen K (1997). Nitrification and denitrification in the rhizosphere of the aquatic macrophyte Lobelia dortmanna L. Limnol Oceanogr.

[CR66] Saunois M, Stavert AR, Poulter B, Bousquet P, Canadell JG, Jackson RB, Raymond PA, Dlugokencky EJ, Houweling S, Patra PK, Ciais P, Arora VK, Bastviken D, Bergamaschi P, Blake DR, Brailsford G, Bruhwiler L, Carlson KM, Carrol M, Castaldi S, Chandra N, Crevoisier C, Crill PM, Covey K, Curry CL, Etiope G, Frankenberg C, Gedney N, Hegglin MI, Höglund-Isaksson L, Hugelius G, Ishizawa M, Ito A, Janssens-Maenhout G, Jensen KM, Joos F, Kleinen T, Krummel PB, Langenfelds RL, Laruelle GG, Liu L, Machida T, Maksyutov S, McDonald KC, McNorton J, Miller PA, Melton JR, Morino I, Müller J, Murguia-Flores F, Naik V, Niwa Y, Noce S, O’Doherty S, Parker RJ, Peng C, Peng S, Peters GP, Prigent C, Prinn R, Ramonet M, Regnier P, Riley WJ, Rosentreter JA, Segers A, Simpson IJ, Shi H, Smith SJ, Steele LP, Thornton BF, Tian H, Tohjima Y, Tubiello FN, Tsuruta A, Viovy N, Voulgarakis A, Weber TS, van Weele M, van der Werf GR, Weiss RF, Worthy D, Wunch D, Yin Y, Yoshida Y, Zhang W, Zhang Z, Zhao Y, Zheng B, Zhu Q, Zhu Q, Zhuang Q (2020) The Global Methane Budget 2000–2017. Earth Syst. Sci. Data, 12(3),1561–1623. 10.5194/essd-12-1561-2020

[CR67] Schütz H, Seiler W, Conrad R (1989). Processes involved in formation and emission of methane in rice paddies. Biogeochemistry.

[CR68] Shiono K, Ogawa S, Yamazaki S, Isoda H, Fujimura T, Nakazono M, Colmer TD (2011). Contrasting dynamics of radial O2-loss barrier induction and aerenchyma formation in rice roots of two lengths. Ann Bot.

[CR69] Soukup A, Votrubová O, Čížková H (2002). Development of anatomical structure of roots of Phragmites australis. New Phytol.

[CR70] Steffens B, Geske T, Sauter M (2011). Aerenchyma formation in the rice stem and its promotion by H_2_O_2_. New Phytol.

[CR71] Tong S, Kjaer JE, Ogorek P, Pellegrini LL, Song E, Pedersen Z, Herzog M (2023). Responses of key root traits in the genus Oryza to soil flooding mimicked by stagnant, deoxygenated nutrient solution. J Exp Bot.

[CR72] Uga Y, Hanzawa E, Nagai S, Sasaki K, Yano M, Sato T (2012). Identification of qSOR1, a major rice QTL involved in soil-surface rooting in paddy fields. Theor Appl Genet.

[CR73] Uga Y, Sugimoto K, Ogawa S, Rane J, Ishitani M, Hara N, Kitomi Y, Inukai Y, Ono K, Kanno N, Inoue H, Takehisa H, Motoyama R, Nagamura Y, Wu J, Matsumoto T, Takai T, Okuno K, Yano M (2013). Control of root system architecture by DEEPER ROOTING 1 increases rice yield under drought conditions. Nat Genet.

[CR74] Wang Q, Zhou F, Shang Z, Ciais P, Winiwarter W, Jackson RB, Tubiello FN, Janssens-Maenhout G, Tian H, Cui X, Canadell JG, Piao S, Tao S (2019). Data-driven estimates of global nitrous oxide emissions from croplands. Natl Sci Rev.

[CR75] Wrage N, Velthof GL, Van Beusichem ML, Oenema O (2001). Role of nitrifier denitrification in the production of nitrous oxide. Soil Biol Biochem.

[CR79] Yamauchi T, Nakazono M (2022). Mechanisms of lysigenous aerenchyma formation under abiotic stress. Trends Plant Sci.

[CR76] Yamauchi A, Kono Y, Tatsumi J (1987). Quantitative root system rice and maize Japan. J Crop Sci.

[CR77] Yamauchi T, Tanaka A, Inahashi H, Nishizawa NK, Tsutsumi N, Inukai Y, Nakazono M (2019). Fine control of aerenchyma and lateral root development through AUX/IAA- and ARF-dependent auxin signaling. Proc Natl Acad Sci U S A.

[CR78] Yamauchi T, Abe F, Tsutsumi N, Nakazono M (2019). Root Cortex provides a venue for gas-space formation and is essential for plant adaptation to Waterlogging. Front Plant Sci.

[CR80] Zhang Q, Huber H, Beljaars SJM, Birnbaum D, de Best S, de Kroon H, Visser EJW (2017). Benefits of flooding-induced aquatic adventitious roots depend on the duration of submergence: linking plant performance to root functioning. Ann Bot.

